# Effect of Trap Regulation on Vacuum DC Surface Flashover Characteristics of Nano-ZnO/PI Film

**DOI:** 10.3390/polym14173605

**Published:** 2022-09-01

**Authors:** Jiang Wu, Bo Zhang, Tianjiao Li, Yan Du, Wen Cao, Hao Yang

**Affiliations:** 1 School of Electronics and Information, Xi’an Polytechnic University, Xi’an 710048, China; 2 Training Center of State Grid Shanghai Electric Power Company, Shanghai 200082, China

**Keywords:** nanocomposite, polyimide film, trap distribution, DC surface flashover, bilayer model

## Abstract

The operating safety of spacecraft in space environments is closely related to the surface discharging phenomenon of dielectrics such as polyimide (PI) film in solar arrays; moreover, carrier traps in the dielectric can affect its insulation performance. Therefore, to improve the vacuum surface flashover characteristics of PI film by nano modification and reveal the effect of trap distribution on the flashover of PI composite film, first, the original PI and nano-ZnO/PI composite films with different additive amounts (0.5, 1, 2, and 3 wt.%) were prepared by in situ polymerization and their performance was evaluated by the physicochemical properties characterized by methods such as thermogravimetric analysis; second, the surface traps of the original and nanocomposite films were measured and calculated by surface potential decay method, and the carrier mobility was also obtained; finally, the vacuum direct current (DC) surface flashover characteristics and bulk resistivity of all the film samples were measured and analyzed. The experiment results showed that with the increase in the amount of nano-ZnO, both the shallow and deep trap density increased significantly, while the trap energy varied slightly, and the surface flashover voltage also increased obviously. Based on the multi-core model, the increases in the shallow and deep trap density after the introduction of nano-ZnO into the PI matrix was analyzed from the microscopic perspective of the interface. Based on the comparative analysis of the trap distribution and surface flashover voltage characteristics, a bilayer model of vacuum DC surface flashover development was proposed. In the bilayer model, deep traps and shallow traps play a dominant role in the vacuum–solid interface and the inner surface of the dielectric, respectively, and increasing the trap density could effectively inhibit secondary electron multiplication on the surface and accelerate charge dissipation inside the film. Consequently, nano-ZnO can purposefully control the trap distribution, and then improve the flashover characteristics of nano-ZnO/PI composite films, which provides a new approach for improving the spacecraft material safety.

## 1. Introduction

When a spacecraft operates in extreme environments in space, various charged particles such as plasma and high-energy electrons are incident on the surface of the spacecraft, resulting in a radiation effect on the dielectric materials, electronic devices, and even on the organisms on the spacecraft [1,2]. Due to the high resistivity and weak ability of charge dissipation, long-term space radiation can cause surface charging followed by serious surface discharging phenomena for the dielectrics, which threaten the spacecraft safety [3,4]. As a type of surface dielectric material widely used in spacecraft, polyimide (PI) film exhibits strong insulation performance, good temperature resistance characteristics, radiation resistance, aging resistance, etc. [5]. Therefore, increasing the vacuum direct current (DC) surface flashover voltage of PI film is one of the key technical approaches to realize future high-voltage and high-power spacecraft with high efficiency and long service life.

Notably, the vacuum DC surface flashover is a complex physical phenomenon, and many contemporary scientists are focusing on its development mechanism [6,7]. Currently, the secondary electron emission avalanche (SEEA) model proposed by Anderson is the most widely used model under the condition of surface flashover in vacuum solid media [8,9]. However, the electron-triggered polarization relaxation (ETPR) model proposed by Blaise and Gressus [10] is used relatively less frequently. Most scholars have basically the same opinions on the initial stage and flashover stage in the flashover process, i.e., that the vacuum surface flashover starts from the electron emission (field-induced electron emission or thermionic emission) at the triple junction where the cathode, insulator, and vacuum meet, and finally form a penetrating discharging channel in the gas desorption layer on the surface of the insulator. However, researchers have different perspectives about the intermediate development stage of flashover. After extensive theoretical research and experiments, the factors that affect the flashover development, such as voltage type [11], electrode structure and material [12], secondary electron emission yield [13], surface state [14], temperature [15], radiation [16], degassing [17], and surface charge [18], have been continuously explored and analyzed. Moreover, it is believed that the charge transportation characteristic of the dielectric is one of the most important factors affecting the flashover development. Therefore, further research on the effect of trap distribution on the trapping and de-trapping processes of charges is expected to reveal the relationship between the charge transport and flashover property, and finally, effectively improve the flashover voltage.

In order to improve the surface flashover characteristics of polymers from the perspective of materials, some scholars have proposed that the addition of a certain amount of nanoparticles into the polymer matrix results in the introduction of a large number of shallow and deep traps. Feng et al. [19] prepared PI/TiO_2_ nanocomposite films with different additive concentrations and found that TiO_2_ could enhance the space charge polarization of the PI matrix at low frequencies and reduce polymer chain motion in orientation polarization to change the dielectric properties of the material. Yang et al. [20] prepared a PI/Cr_2_O_3_ nanocomposite coating, and the introduced traps by nanoparticles could reduce the secondary electron emission yield of PI, and thus improved the surface flashover characteristics. Bhutta et al. [21] studied the electrical conductivity properties of PI/SiO_2_ composites and demonstrated the occurrence of complex electrical conductivity mechanisms in the composite material. Wang et al. [22] studied the vacuum surface flashover characteristics of POSS/PI composites, and believed that a reduction in the resistivity of the composites and trap energy level accelerated the rate of surface charge dissipation, thereby increasing the flashover voltage.

However, some experimental and theoretical research in this field is still required. Initially, few people use nano zinc oxide (ZnO) to modify PI films. Moreover, ZnO is a semiconductive additive with nonlinear electrical conductivity; therefore, it can effectively improve the charge dissipation performance of PI. On the other hand, in the field of mechanistic research, at present, most researchers focus on using SEEA or ETPR models to explain the influence of micro- or nanoparticles on the development of surface flashover in composites. Therefore, from the perspective of a more microscopic level of trap distribution, optimized theories to explain nanoscale controlling effects of additive on the surface flashover voltage of PI film are still lacking.

In this study, the original and nano-ZnO modified PI composite films were prepared using an in situ polymerization method, and the trap distribution was performed for all film samples by isothermal surface potential decay (ISPD) and vacuum DC surface flashover measurement. The relationship between the surface flashover voltage and trap distribution under different additive amounts was studied. Finally, the regulation mechanism of trap distribution on surface flashover was discussed from the perspective of the flashover development, and a bilayer model for flashover development was discussed.

## 2. Experimental Preparation

### 2.1. Preparation Technology of PI Nanocomposite Film

The ZnO/PI nanocomposite film was prepared by in situ polymerization. Detailed information of the raw materials used in the experiment is presented in Table 1.

Notably, aggregation effects of ZnO in the PI may influence the trap distribution properties; therefore, ZnO particles were functionalized by a silane coupling agent (KH550) prior to their modification. First, ZnO powder was dried at 300 °C; then, a certain amount of silane coupling agent was added to an anhydrous ethanol solution (absolute ethanol:deionized water = 9:1). Subsequently, ZnO powder containing particles with a median size of 20 nm was added to the solution, which was then subjected to oscillation via ultrasonication, and the solution was sheared in a water bath at 80 °C. Finally, the solution was filtered and dried to obtain the surface-functionalized-ZnO nanoparticles, which were denoted as KH550-ZnO.

**Figure 1:**
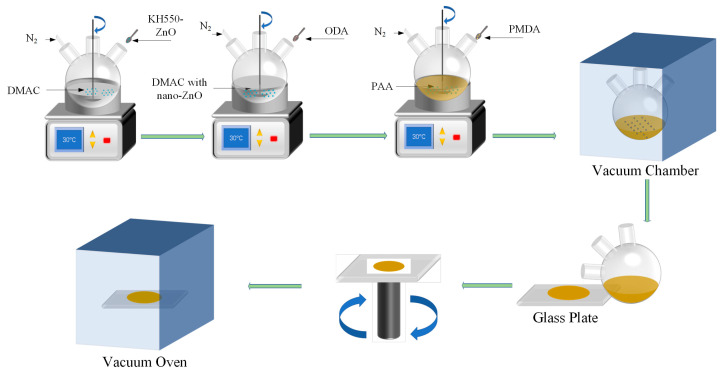
Preparation process of the nano-ZnO/PI film sample.

The composite sample was prepared as follows: First, pyromellitic dianhydride (PMDA) and 4,4′-diaminodiphenyl ether (ODA) were vacuum-dried at 170 and 120 °C for 6 h, respectively. Second, KH550-ZnO powder was added to the *N*,*N*-dimethylacetamide (DMAC) solution in a three-necked flask, and the solution was then mechanically stirred and ultrasonically shaken for 1 h. Then, ODA and PMDA were added successively to the mixed solution. In this step, PMDA was added several times to the solution to obtain a pale yellow viscous polyamic acid (PAA)/nanoparticle slurry, which was then vacuumed and spread on a clean glass plate. Finally, the glass plate was placed in a vacuum oven (Jie Cheng Experimental Apparatus Inc., Shanghai, China), and a thermal imidization process was carried out using a classical gradient heating method at 80 °C for 0.5 h, 120 °C for 0.5 h, 150 °C for 1 h, 250 °C for 1 h, and 300 °C for 1 h [23]. A schematic illustration of the preparation process is presented in Figure 1. The PI nanocomposite films with different mass fractions of ZnO were prepared and labelled as the original PI, 0.5 wt.% ZnO/PI, 1 wt.% ZnO/PI, 2 wt.% ZnO/PI, and 3 wt.% ZnO/PI. The thickness of the films was about 60 μm, with a diameter about 100 mm.

### 2.2. Vacuum DC Surface Flashover Characteristic Measurement System

Before the surface flashover test, the sample surface was ultrasonically cleaned with absolute ethanol for 30 min, then degassed in a vacuum oven for 24 h, and finally placed in a drying dish for 12 h at room temperature.

**Figure 2:**
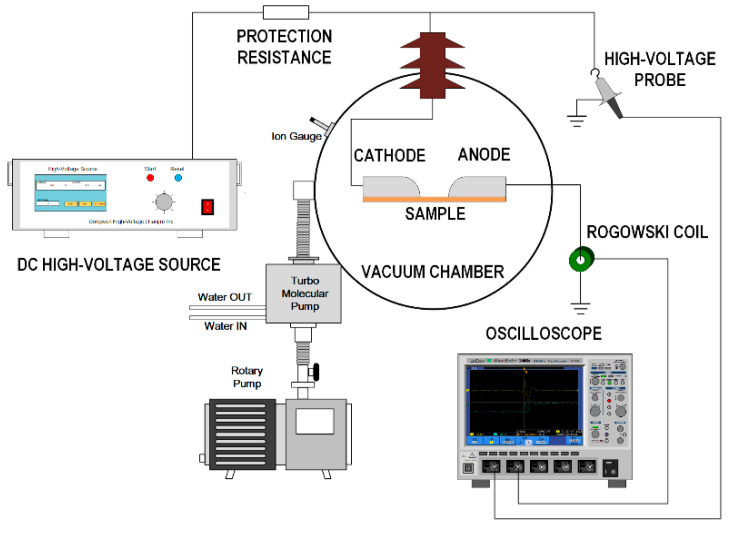
Vacuum DC surface flashover voltage measurement system.

The vacuum DC surface flashover measurement system is shown in Figure 2. In the surface flashover measurement system, a finger-shaped electrode was utilized in a high-vacuum environment (10^−5^ Pa), and the distance between the cathode and the anode was 1 mm. The measurement system consisted of a negative 30 kV DC high-voltage source (Dong Wen High-Voltage Power Supply Corp., Tianjin, China), 1000:1 high voltage probe (Tektronix Inc.(China), Shanghai, China), Rogowski coil (Pearson Electronics Inc., Palo Alto, USA), and oscilloscope (Tektronix Inc.(China), Shanghai, China). The high-voltage probe was used to measure the voltage signal, while the Rogowski coil was used to measure the flashover current signal. The measured voltage and current dual-channel signals were then collected to the oscilloscope, and the test results were finally obtained. The test was carried out at room temperature, and the voltage rising rate was 100 V s^−1^. To obtain stable results, the surface flashover voltage was measured continuously more than 30 times with a measurement time interval of 60 s.

### 2.3. Surface Trap Distribution Measurement System

**Figure 3:**
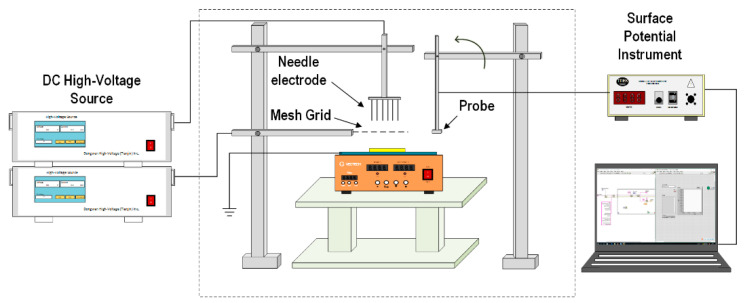
Isothermal surface potential decay measurement system.

The ISPD method can be used to obtain the apparent trap distribution by measuring the decay behavior of the surface deposited charges under isothermal conditions [24,25]. Figure 3 displays the surface potential decay experimental system based on the corona charging method. The corona charging electrode consisted of a needle electrode and a mesh grid electrode, and the distance between the mesh grid and sample surface was 5 mm. Both the needle electrode and the mesh grid electrode were connected to a DC high-voltage source, and the sample back electrode was grounded. The electrostatic potentiometer (Advanced Energy Industries Inc., Calabasas, USA) was a TREK 347B model, and the probe model (Advanced Energy Industries Inc., Calabasas, USA) was TREK-6000B-14. During the experiment, a transparent testing box was used to ensure the relative stability of the temperature and humidity. The sample was placed under the mesh grid electrode for corona charging for 15 s; next, the probe was quickly moved directly above the sample. Finally, the experimental data of surface potential decay curve were recorded with a computer system.

The process of charge injection and migration is closely related to the microscopic dynamic process. Charges deposited on the dielectric surface are usually dissipated in three ways, i.e., ion neutralization with the surrounding gas, dissipation along the dielectric surface, and dissipation through the dielectric volume to the back electrode. The experimental humidity was controlled at about 30% and the mesh grid electrode was used to make the surface potential uniform; therefore, the electric field in the horizontal direction of the sample was considered to be zero. Thus, the dissipation process of the surface charge only considered the volume dissipation through the dielectric. Moreover, in this study, it was also assumed that the de-trapped charges would not be trapped again. Based on the abovementioned assumptions, through the theory of Simmons, the calculation formula of trap energy and trap density could be obtained [26,27] as follows:

(1) $$E_{t}=E_{m}-E_{V}=k_{B}T\ln(vt)$$

(2) $$N(E_{t})=\frac{4\varepsilon_{0}\varepsilon_{r}}{qL^{2}k_{B}T}\left|t\frac{dV_{s}(t)}{dt}\right|$$

where *E_t_* is the trap energy, *T* is the absolute temperature, *k_B_* is the Boltzmann constant, *t* is the potential decay time, *N*(*E_t_*) is the trap density, *V_s_*(*t*) is the surface potential of the sample, *L* is the sample thickness, and *v* is the escape frequency. The measured surface potential decay data conformed to the decay law of the double exponential function, and the experimental dataset was fitted with a double exponential function. The fitting formula is as follows:

(3) $$V_{s}(t)=A_{1}e^{-t/\tau_{1}}+A_{2}e^{-t/\tau_{2}}$$

where *A*_1_, *A*_2_, *τ*_1_, and *τ*_2_ are fitting parameters. These equations were used to calculate the trap energy and trap density of the dielectric. Moreover, the migration of space charges in the dielectric followed the basic laws of electromagnetism. According to the Poisson equation and the current continuity equation, combined with the surface potential decay rate, the carrier mobility can be calculated [28] as follows:

(4) $$\left|\frac{dV(t)}{dt}\right|=\frac{d^{2}}{2\mu t^{2}}$$

(5) $$\mu =\frac{d^{2}}{t_{T}V_{0}(t)}$$

where *t_T_* is the time required for the space charge to migrate from the surface to the back electrode, i.e., the transmission time, and *V*_0_(*t*) is the initial potential. An inflection point occurs at *t_T_* in the process of charge migration, and the mobility *μ* is obtained from Equation (5).

### 2.4. Bulk Resistivity Measurement

The bulk resistivity of the sample was measured using a picoammeter (type Keithley 6517, Tektronix Inc., Beaverton, ON, USA) with electrode testing box (type 8009, Tektronix Inc., Beaverton, ON, USA), and the maximum measurement voltage was DC 1000 V. In this experiment, the test temperature was 300 K, and each test lasted for 15 s. To ensure the accuracy of the experiment, 8 data points were collected for each sample, and the resistivity value and experimental error were calculated.

## 3. Experimental Results and Analysis

### 3.1. Performance Characterization of ZnO/PI Films

The basic physicochemical properties of the original and nanocomposite PI films were characterized by surface morphological analysis, functional group analysis, and thermal stability analysis.

**Figure 4:**
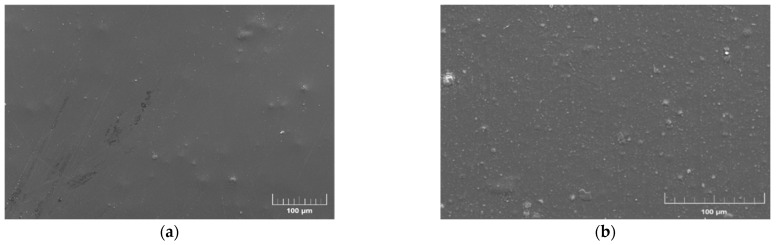
Representative SEM image of PI films. (**a**) Surface of the original PI film; (**b**) surface of the 2 wt.% ZnO/PI composite film.

In order to efficiently utilize the inorganic–organic interface for trap regulation, the dispersion property of the nano-ZnO particles in PI is important, and the sample surface was characterized by scanning electron microscopy (SEM). Figure 4a,b exhibits the representative SEM images of original PI and 2 wt.% ZnO/PI composite film, respectively. Comparative analysis of the figures indicates that the ZnO particles, evidently shown as the light spots, are basically evenly distributed on the surface of the 2 wt.% ZnO/PI composite. In contrast, the original PI shows a slight spot, which possibly correspond to other impurities. The results indicate the strong compatibility of the ZnO filler with the PI matrix.

**Figure 5:**
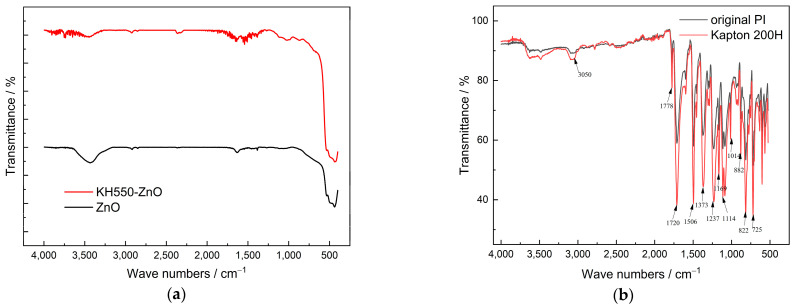
Infrared spectrum of ZnO/PI and PI films. (**a**) Untreated ZnO and KH550-ZnO; (**b**) original PI and commercial Kapton film.

Compared with the raw ZnO particles, the KH550-ZnO was considered to possess a better dispersion property in the PI matrix. Fourier-transform infrared (FTIR) spectroscopy was performed to verify the surface functionalization of ZnO. The measurement wavelength was in the range of 4000–400 cm^−1^, and the measured IR spectrum is shown in Figure 5a. The absorption peaks of untreated ZnO appear at around 470 and 3440 cm^−1^, corresponding to the stretching vibration peak of the Zn–O bond and the stretching vibration peak of –OH, respectively. After KH550 surface treatment, the characteristic peak of Zn–O bond still existed, while the vibration peak of –OH weakened, which indicates that the coupling agent reacts with –OH on the surface of ZnO. Furthermore, the peaks near 3700 and 1500 cm^−1^ may be attributed to the vibration peaks of –NH and –CH_2_ in KH550, which further demonstrates the reaction between the coupling agent and ZnO. 

Simultaneously, the typical functional groups of PI were compared between original PI film and commercial Kapton film (type Kapton 200H), as shown in Figure 5b. The peak at 3050 cm^−1^ corresponds to the stretching vibration of =CH on benzene ring, the peak at 1778 cm^−1^ is attributed to the isophase or symmetric stretching vibrations of two carbonyl groups on a pentaimine ring, the peak at 1720 cm^−1^ corresponds to the inverse or asymmetric stretching vibrations of two carbonyl groups on a pentaimine ring, and the peak at 1506 cm^−1^ can be attributed to the C–C bond peak of benzene ring. Moreover, the peak at 1373 cm^−1^ is attributed to the C–N stretching vibration; that at 1237 cm^−1^ is ascribed to telescopic vibration of =C–O–C=; the peaks at 1169, 1114, 1014, 882, and 822 cm^−1^ are related to benzene ring, and the peak at 725 cm^−1^ corresponds to =C–H rocking. These peaks were confirmed in the results, which indicates the successful sample preparation without the formation of any intermediate products such as PAA.

**Figure 6:**
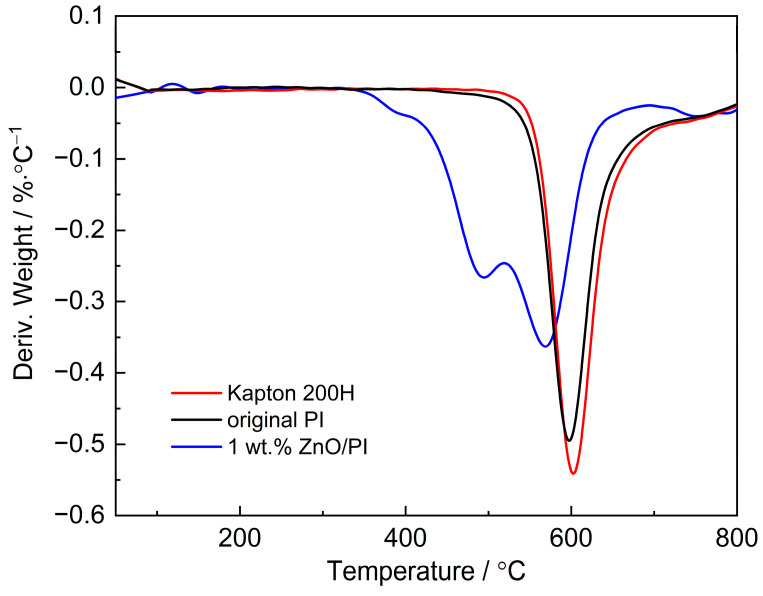
DTG curves of original PI and nano-ZnO/PI film.

According to the application under high temperature in space environments, the thermal properties should be verified for the prepared composite film. Thermogravimetric analysis (TGA) under a nitrogen atmosphere was carried out, and the temperature was set from 50 to 800 °C with a heating rate of 10 K min^−1^. The temperature of 5% weight loss (T_5_) of commercial Kapton film (type Kapton 200H) was at 538.8 °C, whereas the T_5_ values of the prepared original PI film and 1 wt.% ZnO/PI film were 570.5 and 454.83 °C, respectively, which indicates a well thermal stability at high temperature. Furthermore, the thermal decomposition rate was obtained using a differential TG (DTG) curve by performing a differential calculation on the TGA curve, as shown in Figure 6. The maximum thermal decomposition rate was used to evaluate the thermal stability of the PI films.

Figure 6 illustrates that the original PI film started to decompose at around 500 °C and the decomposition rate was the highest at 597.5 °C, which approximated that of the Kapton film at 602.5 °C. It was speculated that for the ZnO-modified film, the thermal imide cyclization of PAA may be incomplete, which led to slight weakening in thermal properties; as a result, the temperature of maximum thermal decomposition rate of 1 wt.% ZnO/PI film dropped to 575.5 °C.

In summary, the original PI and nanocomposite PI films prepared in this study exhibited excellent thermal properties, and the nanocomposite PI film is a suitable candidate dielectric material for applications in extreme space environments.

### 3.2. Vacuum DC Surface Flashover Characteristics of ZnO/PI Composite Films

**Figure 7:**
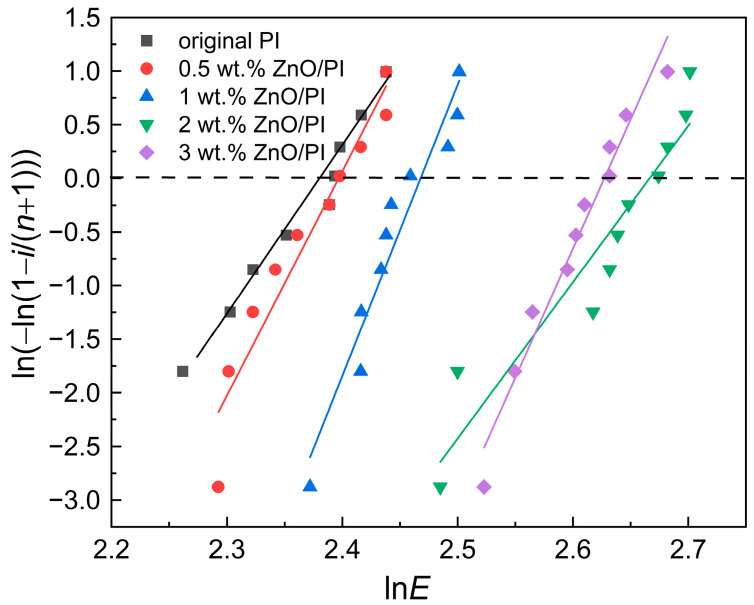
Weibull statistical distribution of the DC surface flashover voltage of ZnO/PI composite films.

The multiple measurement results of the vacuum DC surface flashover voltage showed a certain dispersion; therefore, the two-parameter Weibull statistical distribution method was used in the data analysis, and the results are shown in Figure 7. The data distribution characteristic of each sample was represented by the shape parameter *β*, whereas the average value of DC surface flashover voltage was represented by *α*. The calculation results are presented in Table 2.

Table 2 shows that the average surface flashover voltage clearly increased with the increase in the amount of ZnO additive. When the amounts of ZnO were 0.5 and 1 wt.%, the flashover voltage increased by 3.56% and 11.19%, respectively, compared with the original PI film. When the amount of ZnO was 2 wt.%, the surface flashover voltage increased by 35.63% and reached the highest. However, when the amount of ZnO was 3 wt.%, the increasing rate of the flashover voltage decreased slightly to 30.41%. The experimental results indicate that the amount of nano-ZnO effectively influenced the surface flashover voltage.

### 3.3. Surface Potential Decay Characteristic of Nano-ZnO/PI Films

**Figure 8:**
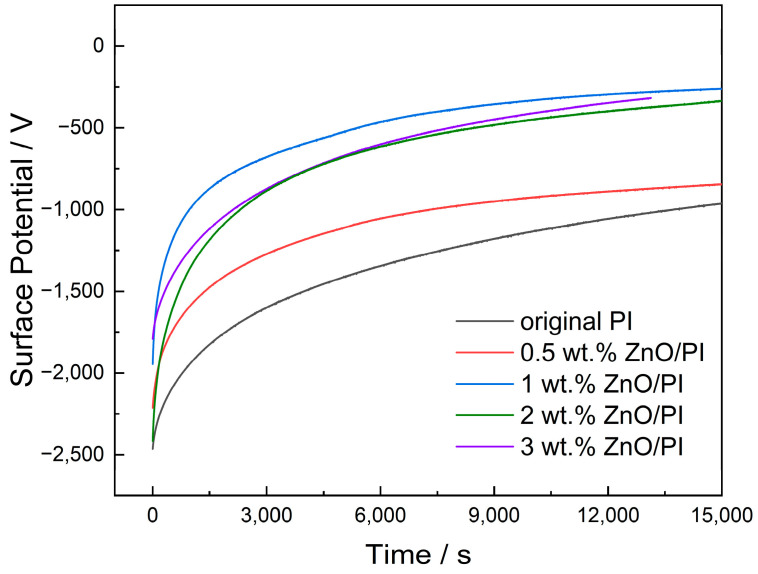
Surface potential decay curves under negative corona charging conditions.

Figure 8 shows the surface potential decay curve of the film samples under negative corona charging conditions. With the increase in the amount of ZnO, the initial potential decreased continuously, and the balanced potential showed a trend of first decreasing and then increasing. The surface potential was the lowest when the amount of ZnO was 1 wt.%. The decreasing surface potential indicates that the nanoparticle accelerated the dissipation of surface charges.

Initially, the first 2000 s marked the rapid decay of the surface potential. The induced electrostatic field between the upper surface of the sample and the back electrode was large, and the charges bound by the shallow traps were easily de-trapped and migrated to the back electrode. As the time progressed, the charges in the shallow traps became less; nonetheless, it was more difficult for the charge to escape from the deep trap. Therefore, the surface potential exhibited a nonlinear decay phenomenon that was fast at first and then slowed down.

## 4. Discussion

### 4.1. Surface Trap Distribution of Nano-ZnO/PI Films

**Figure 9:**
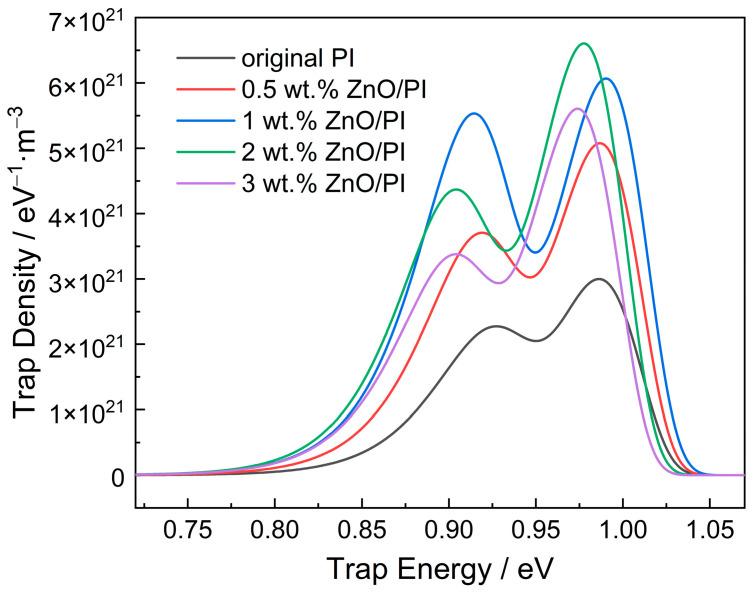
Surface trap distribution of nano-ZnO/PI with different amounts of additive.

After fitting with a double exponential function as described in Section 2.3, the relationship between the surface trap energy and trap density of the original PI and nanocomposite films with different amounts of ZnO was obtained, as shown in Figure 9. The trap distribution curve exhibits the existence of two distinct peaks of trap density, which represent a shallow electron trap and deep electron trap, respectively. The shallow trap energy is distributed between 0.85 and 0.95 eV, whereas the deep trap energy is between 0.92 and 1.03 eV. The deep trap density is significantly higher than that of shallow trap.

With increasing amounts of nanoparticles, the shallow trap energy shows a slightly decreasing trend, and the shallow trap density first increases and then decreases as shown in Table 3. The shallow trap density peaks at 1 wt.% of ZnO, which is 5.532 × 10^21^ eV^−1^·m^−3^. The deep trap energy has no obvious change with the increase in the amount of nano-ZnO; however, the deep trap density also shows a trend of first increasing and then decreasing, which is similar to that of the shallow trap. When the amount of ZnO is 2 wt.%, the deep trap density reaches a peak value of 6.603 × 10^21^ eV^−1^·m^−3^.

**Figure 10:**
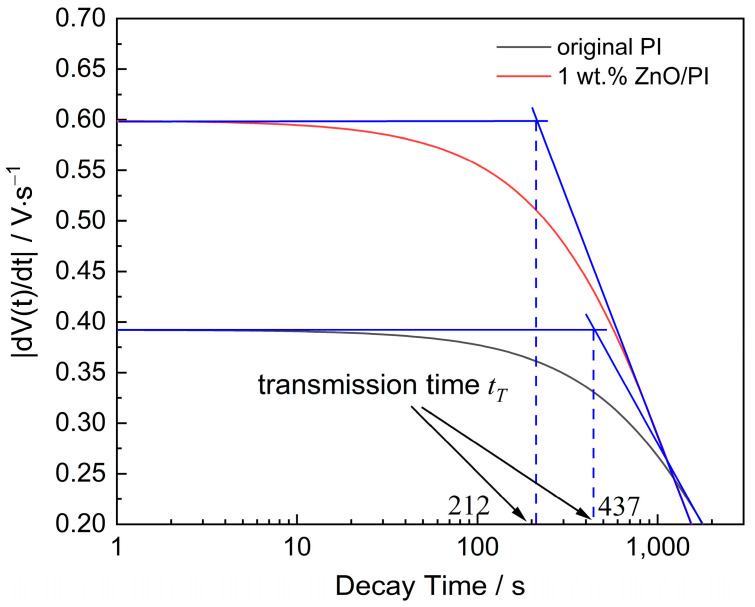
The surface potential decay rate of original PI and 1 wt.% ZnO/PI.

Figure 10 shows the surface potential decay rate of the original PI film and 1 wt.% ZnO/PI composite film, and the transit time was obtained for carrier mobility based on Equation (5). The calculation results of carrier mobility are listed in Table 4. The results indicate that with the increase in the amount of ZnO, the carrier mobility of the composite film continuously increases.

### 4.2. Relationship between Trap Distribution and DC Surface Flashover Voltage

**Figure 11:**
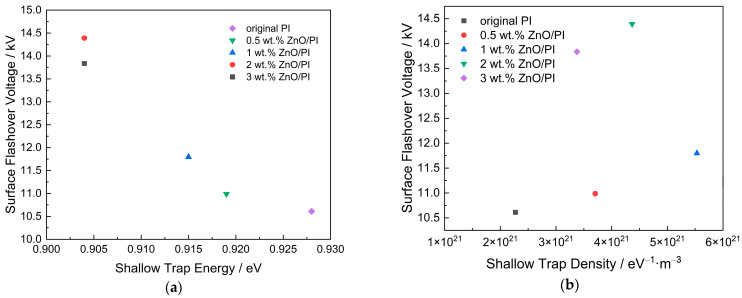

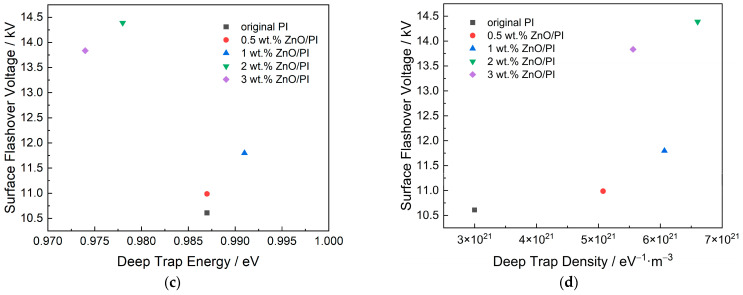
Relationship between surface trap distribution and surface flashover voltage. (**a**) Variation in flashover voltage with shallow trap energy; (**b**) variation in flashover voltage with shallow trap density; (**c**) Variation in flashover voltage with deep trap energy; (**d**) Variation in flashover voltage with deep trap density.

Combining the trap distribution and the DC surface flashover voltage, the surface flashover voltage varies with the shallow and deep trap characteristics, as shown in Figure 11.

Figure 11 demonstrates that with the decrease in the shallow trap energy, the surface flashover voltage shows an obvious upward trend, whereas the increase in the shallow trap density also causes the flashover voltage to fluctuate and eventually rise. On the other hand, the surface flashover voltage also increases significantly with the increase in deep trap density. The deep trap energy varies slightly as shown in Table 3; thus, the influence of the deep trap energy on the flashover voltage has no obvious regularity.

### 4.3. The Regulation Mechanism of Trap Distribution on the Surface Flashover Development

#### 4.3.1. Effects of Nanoparticles on the Trap Distribution of Composite Film

**Figure 12:**
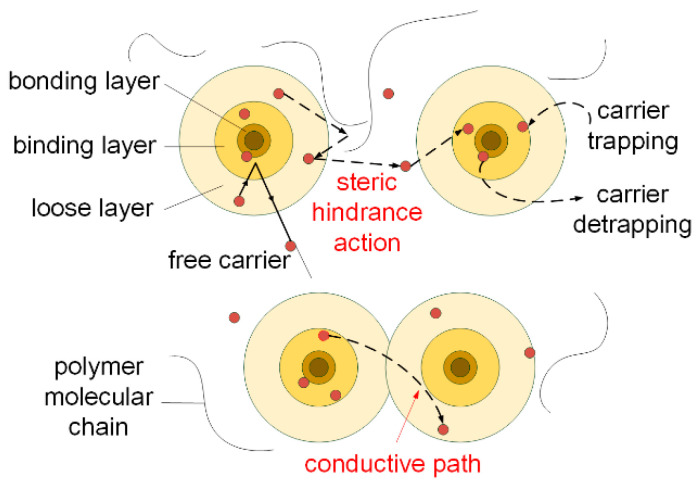
Carrier transport process in nano-ZnO modified polymers based on the multi-core model.

Based on the multi-core model proposed by Tanaka et al. [29], the transport process of carriers inside the polymer was analyzed, as shown in Figure 11. In the multi-core model, after the polymer matrix is modified with nanoparticles, new chemical bonds are generated between the polymer matrix and the nanoparticles. The interface between the nanoparticles and the matrix material forms a loose layer, a binding layer, and a bonding layer from outside to inside, which leads to the introduction of a large number of deep and shallow traps, and the carrier mobility shows an upward trend. When the amount of nanoparticles is lower, compared with the original polymer matrix, the interface area formed by the nanoparticles modified with the coupling agent increases; moreover, the steric hindrance of the nanoparticles effectively blocks the charge transport inside the polymer. However, with the continuous increase in the amount of nanoparticles to a certain level, the overlapping phenomenon gradually appears in the interface area, as shown in Figure 12, and a conductive path is formed in the overlapping area, which accelerates the transport process of carriers.

For the nano-ZnO/PI composite films, when the amount of nano-ZnO is 1 wt.%, the number of shallow traps in the dielectric surface is the largest, and carriers are easily de-trapped from the shallow traps to participate in the charge transport process. With the continuous increase in the amount of nano-ZnO, the density of deep traps increases, and the deep traps can effectively inhibit the charge transport process when the amount of nano-ZnO is 2 wt.%. In contrast, when the amount of nano-ZnO reaches 3 wt.%, the interface overlap phenomenon occurs; thus, the trap density decreases slightly and a new conductive path appears for charge transport.

**Figure 13:**
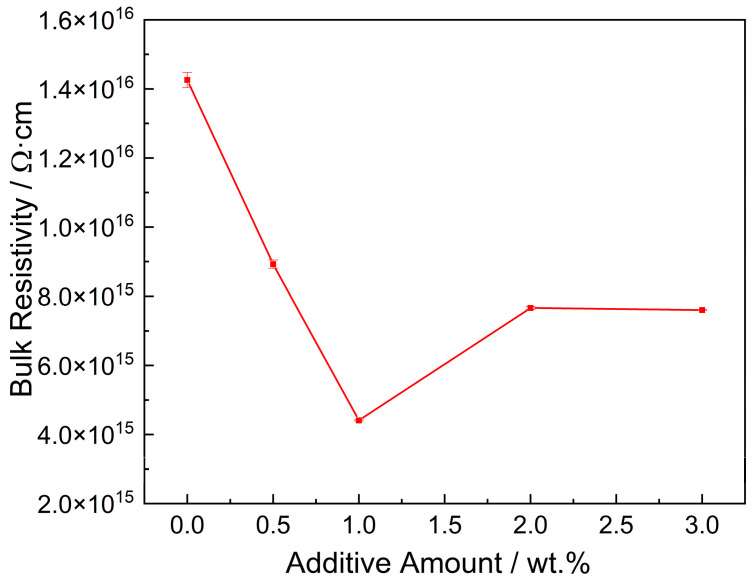
Bulk resistivity of PI films with different amounts of nano-ZnO.

Based on the abovementioned regulation mechanism of trap distribution, the nano-ZnO particles change the charge transport properties of the nanocomposite films, which can be reflected in the macroscopic property of bulk resistivity. Figure 13 shows that the bulk resistivity of the original PI film is the highest, which is 1.43 × 10^16^ Ω·cm. With the increase in the amount, the bulk resistivity shows a trend of first decreasing and then increasing, and the peak value appears when the mass fraction is 1 wt.%. At the lowest point, the bulk resistivity of the composite film is 4.41 × 10^15^ Ω·cm.

#### 4.3.2. Influence of Trap Distribution on Surface Flashover Development

According to the traditional SEEA model, the surface flashover develops from the triple junction with a high local electric field. The electrons are excited by the field emission and bombard the surface of the dielectric; then, the secondary electrons are generated. The generated secondary electrons are directed to the anode under the electrostatic field. During the electron movement, the secondary electrons continue to bombard the dielectric surface to generate more new secondary electrons, accompanied with dielectric gas desorption. Development of this process occurs from cathode to anode, and eventually forms an electron avalanche in the surface, which finally leads to surface flashover [8]. On the other hand, according to the ETPR model, negative and positive charge accumulation inside the dielectrics easily leads to polarization regions, and the polarization regions interact to form a strong electrostatic field and maintain an equilibrium. However, the trapped charges are de-trapped when disturbed by external light, electricity, heat, or even mechanical stress, and the polarization balance is destroyed, resulting in polarization relaxation of the dielectric to release polarization energy. This polarization energy can stimulate more electrons to escape from the traps, resulting in surface degassing, and eventually, surface flashover [10].

**Figure 14:**
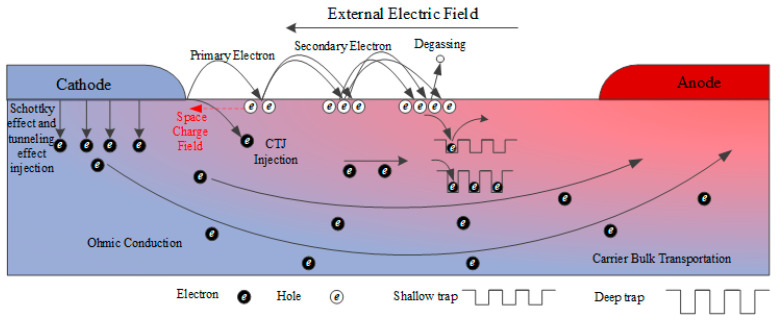
Bilayer model for the development of vacuum DC surface flashover.

By integrating the two abovementioned flashover models, the effect of trap distribution on the development of surface flashover is divided into following two parts: the electron multiplication on dielectric surface and the change transport inside the dielectric surface. Based on the influence of traps on charge transport, a bilayer model for the surface flashover development was established, as shown in Figure 14.

The first layer is the vacuum–solid interface layer along the surface. For the original PI, with a continuous increase in the applied voltage, the emitted primary electron of the Schottky effect increases exponentially at the triple junction of the cathode–vacuum–dielectric. The primary electron then leads to the multiplication of the secondary electrons on the dielectric surface, eventually producing a penetrating flashover channel. After the dielectric was modified with nano-ZnO, the density of deep traps on the surface of the material increased; therefore, the excited electron in the dielectric by the injected electron is easily captured by the deep trap and it is difficult to de-trap. Therefore, the probability of secondary electron emission is considered to reduce, which weakens the flashover development process. In this case, more primary electrons are only generated from the cathode to form enough secondary electrons and promote the surface flashover development; thus, a higher applied voltage on the cathode is required.

The second layer is the inner surface layer of the dielectric. When the emitted electrons from cathode are injected into the surface of the dielectric, a portion of the electrons migrates to the interior of the dielectric through bulk transport. Some of the charges are captured by the traps; thus, a positive and negative charge center is easily formed inside, generating an equilibrated polarized region. Influenced by the external electric field disturbance, the equilibrium state of the polarized region is disturbed and relaxation polarization occurs, resulting in surface flashover. The charge transport in the dielectric is closely related to the trap distribution; therefore, the shallow traps are especially beneficial to the charge transport. The abovementioned results indicate that the density of shallow traps increases with the nano-ZnO additive, along with the increase in carrier mobility, which leads to an increase in bulk conductivity. Therefore, a large number of charges in the original polarization region can dissipate rapidly, and the relaxation polarization effect is then weakened under the disturbance of the external electric field; as a result, the flashover voltage increases.

The bilayer model of vacuum DC surface flashover comprehensively expounds the flashover development process of nano-modified PI from the vacuum–solid interface and solid surface layer. Compared with the traditional SEEA model and ETPR model, the bilayer model clearly explains the effect of surface traps on the development of flashover, and pushes the previous theoretical research work to a more microscopic level. Moreover, analyzing the trap regulation mechanism of the surface flashover leads to an active improvement method of flashover voltage, which provides a theoretical basis for the subsequent selection of other types of additives. The research reported herein indicates that the method of nano-ZnO modified PI film to improve its flashover voltage is effective. However, we should clearly recognize that, compared with the original PI film, the production of the composite film has become more complex. For example, the agglomeration of ZnO particles may instead reduce the dielectric properties such as bulk resistivity and breakdown strength. Moreover, compared with the original PI film, the brittleness of the ZnO/PI film also increases. Furthermore, studies on the stability of the trap regulation method need systematic explorations to verify it under extreme space environments, particularly, under the influence of high-energy electron radiation, high–low temperature cyclic aging, and other factors.

## 5. Conclusions

The PI composite films with different amounts of nano-ZnO were prepared by in situ polymerization. The physicochemical properties and thermal stability of all film samples were then verified. Based on the results of this study, following conclusions can be drawn:

(1) The nano additive can influence the surface trap distribution of the nano-ZnO/PI film and thus affect the surface charge transport. With the increase in the amount of nano-ZnO, the deep trap energy of ZnO/PI film varies slightly, whereas the shallow trap energy decreases slightly. Moreover, both shallow and deep trap density show a trend of first increasing and then decreasing, and carrier mobility increases continuously.
(2) The vacuum DC surface flashover voltage of the nano-ZnO/PI composite film clearly increases. The flashover voltage continues to rise with the increase in the amount of ZnO and reaches a peak for 2 wt.% ZnO/PI, and then the flashover voltage decreases slightly with the further increase in additive amount. Comparative analysis of the trap distribution and flashover voltage mainly concludes that the increases in shallow and deep trap density both contribute to the rising of flashover voltage.
(3) The regulation effect of trap distribution on the vacuum DC surface flashover development was analyzed using a bilayer model. The results of the bilayer model indicate that on the vacuum–solid surface of the nanocomposite film, deep traps dominate. The increase in the deep trap density can effectively suppress the multiplication of secondary electrons. Moreover, in the inner surface of the dielectric, shallow traps dominate. The decreased shallow trap energy and increased trap density improve the dissipation speed of the surface charge, which is beneficial to the increase in the flashover voltage.
(4) The trap distribution characteristics of the nano-ZnO/PI composite film exhibit an obvious effect on the improvement in the flashover voltage. Therefore, in future studies, it is necessary to verify its effectiveness under extreme space environments such as high-energy electron radiation and high–low temperature cyclic aging.

## Figures and Tables

**Table 1:** Raw materials for the preparation of the PI film.

Material	CAS	Company	Purity
Silane coupling agent (KH550)	919-30-2	Aladdin Inc. (Shanghai, China)	99%
Anhydrous ethanol	64-17-5	Aladdin Inc. (Shanghai, China)	moisture content ≤0.3%
Nano ZnO	1314-13-2	ZHONGKEJINYAN Inc. (Beijing, China)	99.98%
*N*,*N*-Dimethylacetamide	127-19-5	Aladdin Inc. (Shanghai, China)	≥99.8%
Pyromellitic dianhydride	89-32-7	Aladdin Inc. (Shanghai, China)	99%
4,4′-Diaminodiphenyl ether	101-80-4	Aladdin Inc. (Shanghai, China)	99%

**Table 2:** Weibull distribution parameters of surface flashover characteristic of ZnO/PI composite films.

Parameter	Original PI	0.5 wt.% ZnO/PI	1 wt.% ZnO/PI	2 wt.% ZnO/PI	3 wt.% ZnO/PI
*β*	14.842	20.898	27.138	14.575	24.076
*α*	10.610	10.988	11.797	14.390	13.836
error	1.106	1.259	1.258	1.343	1.185

**Table 3:** Surface trap energy and density of original PI and nanocomposite films.

Samples	Shallow Trap Energy/eV	Shallow Trap Density/eV^−1^‧m^−3^	Deep Trap Energy/eV	Deep Trap Density/eV^−1^‧m^−3^
original PI	0.928	2.273 × 10^21^	0.987	2.998 × 10^21^
0.5 wt% ZnO/PI	0.919	3.706 × 10^21^	0.987	5.076 × 10^21^
1 wt% ZnO/PI	0.915	5.532 × 10^21^	0.991	6.067 × 10^21^
2 wt% ZnO/PI	0.904	4.368 × 10^21^	0.978	6.603 × 10^21^
3 wt% ZnO/PI	0.904	3.378 × 10^21^	0.974	5.604 × 10^21^

**Table 4:** The carrier mobility variation in composite films with different amounts of nano-ZnO.

Sample	Transmission Time *t_T_*/s	Thickness *d*/μm	Mobility *μ*/m^2^·(V·s)^−1^
Original PI	437	58	3.12 × 10^−15^
0.5 wt.% PI/ZnO	344	52	3.55 × 10^−15^
1 wt.% PI/ZnO	212	50	6.06 × 10^−15^
2 wt.% PI/ZnO	283	67	6.56 × 10^−15^
3 wt.% PI/ZnO	209	58	8.98 × 10^−15^

## Data Availability

The data used to support the findings of this study are available from the corresponding author upon request.

## References

[1] Abdel-Aziz Y.A., Abd EI-Hameed A.M., Ismail M.I., Ahmed A., Elifiky D., Gregorio A. (2021). Effects of space plasma on an oxide coating of spacecraft’s surface materials. Adv. Space Res..

[2] Polsak A.W., Delacourt B., Disa N., Semprimoschnig C.O.A. (2019). Material charging investigations for solar orbiter. IEEE Trans. Plasma Sci..

[3] Cooper R., Ferguson D., Engelhart D.P., Hoffmann R. (2017). Effects of radiation damage on polyimide resistivity. J. Spacecraft Rockets.

[4] Song B.P., Zhou R.D., Yang X., Zhang S., Yang N., Fang J.Y., Song F.L., Zhang G.J. (2021). Surface electrostatic discharge of charged typical space materials induced by strong electromagnetic interference. J. Phys. D-Appl. Phys..

[5] Yaqoob A.A., Safian M.T., Rashid M., Parveen T., Umar K., Ibrahim M.N.M. (2021). Chapter One-Introduction of Smart Polymer Nanocomposites. Smart Polymer Nanocomposites: Biomedical and Environmental Applications.

[6] Li S.T. (2020). Improvement of surface flashover in vacuum. High Volt..

[7] Xing Z., Chen W., Li Z., Xue N., Li F., Dai X., Guo S., Cui H. (2021). Study on high frequency surface discharge characteristics of SiO_2_ modified polyimide film. Polymers.

[8] Anderson R.A., Brainard J.P. (1980). Mechanism of pulsed surface flashover involving electron-stimulated desorption. J. Appl. Phys..

[9] Su G.Q., Wang Y.B., Guo B.H., Song B.P., Mu H.B., Zhang G.J. (2017). Experimental investigation of surface charge accumulation behaviors on PTFE insulator under DC and impulse voltage in vacuum. IEEE Trans. Dielectr. Electr. Insul..

[10] Blaise G., Legressus C. (1991). Charging and flashover induced by surface polarization relaxation process. J. Appl. Phys..

[11] Kuffel E., Grzybowski S., Ugarte R.B. (1972). Flashover across polyethylene and tetrafluoroethylene surfaces in vacuum under direct, alternating and surge voltages of various waveshapes. J. Phys. D-Appl. Phys..

[12] Yamamoto O., Hara T., Nakae T., Hayashi M., Ueno I. (1989). Effects of spark conditioning, insulator angle and length on surface flashover in vacuum. IEEE Trans. Electr. Insul..

[13] Wang W.W., Li S.T., Min D.M. (2016). Enhanced flashover strength in polyethylene nanodielectrics by secondary electron emission modification. AIP ADV.

[14] Yamamoto O., Takuma T., Fukuda M., Nagata S., Sonoda T. (2003). Improving withstand voltage by roughening the surface of an insulating spacer used in vacuum. IEEE Trans. Dielectr. Electr. Insul..

[15] Tu Y., Yi C., Wang S., Qin S., Yuan Z., Fan L. (2019). Effect of temperature on polyimide dc flashover characteristics in different vacuum degrees. J. Phys. D-Appl. Phys..

[16] Wang X., Zheng S., Li Z., Pan S., Fan W., Min D., Li S. (2022). Radiation electron trajectory modulated DC surface flashover of polyimide in vacuum. J. Phys. D-Appl. Phys..

[17] Neuber A.A., Butcher M., Krompholz H., Hatfield L.L., Kristiansen M. (2000). The role of outgassing in surface flashover under vacuum. IEEE Trans. Plasma Sci..

[18] Guan H., Chen X., Du H., Jiang T., Paramance A., Zhou H. (2020). Surface potential decay and DC surface flashover characteristics of DBD plasma-treated silicone rubber. Nanotechnology.

[19] Feng Y., Yin J., Chen M., Song M., Su B., Lei Q. (2013). Effect of nano-TiO_2_ on the polarization process of polyimide/TiO_2_ composites. Mater. Lett..

[20] Yang X., Sun G., Zhou R., Huang K., Li W., Wang C., Dong J., Song B., Zhang G. (2022). Ultralow secondary electron emission and improved vacuum surface insulation of polyimide with scalable nanocomposite coating. Appl. Surf. Sci..

[21] Bhutta M.S., Akram S., Meng P., Castellon J., Agnel S., Li H., Guo Y., Rasool G., Hussain S., Nazir M.T. (2021). Steady-state conduction current performance for multilayer polyimide/SiO_2_ films. Polymers.

[22] Wang J., Xiao R., Liu R., Ping A., Wang Z., Liu J., Zhang S., Liu Y. (2022). DC surface flashover characteristics of polyimide containing polyhedral oligomeric silsesquioxane (POSS) in the main chains under vacuum. Polymers.

[23] Zhai Y. (2007). Controllable aggregation structure preparation and films properties study for polyimide based on PMDA-ODA. Ph.D. Thesis.

[24] Han Y.S., Li S.T., Min D.M. (2018). Trap energy distribution in polymeric insulating materials through surface potential decay method. IEEE Trans. Dielectr. Electr. Insul..

[25] Rychkov A., Kuznetsov A., Gulyakova A., Rychkov D. (2021). Surface potential decay of corona charged polyethylene films: Influence of deep surface traps. IEEE Trans. Dielectr. Electr. Insul..

[26] Simmons J.G., Tam M.C. (1973). Theory of isothermal currents and the direct determination of trap parameters in semiconductors and insulators containing arbitrary trap distributions. Phys. Rev. B.

[27] Chen G., Xu Z. (2009). Charge trapping and detrapping in polymeric materials. J. Appl. Phys..

[28] Lewis T.J. (2004). Interfaces are the dominant feature of dielectrics at the nanometric level. IEEE Trans. Dielectr. Electr. Insul..

[29] Tanaka T., Kozako M., Fuse N., Ohki Y. (2005). Proposal of a multi-core model for polymer nanocomposite Dielectrics. IEEE Trans. Dielectr. Electr. Insul..

